# Intelligence

**Published:** 1831-01

**Authors:** 


### INTELLIGENCE.

### PETITION FOR PRACTICE.

The following printed circular has been recently distributed in a respectable neighbourhood by a medical practitioner. We abstain from publishing the name and address of this petitioner for public patronage; but we must take the liberty of assuring him that such a system cannot promote his own interest, and that it must tend to degrade the respectability of the profession in the estimation of the public. We would advise Mr.——, if he despairs of success by the fair and honourable exercise of his professional abilities, to take up with some inferior calling, the character of which will suffer no disgrace by his publicly advertising the price of his articles and labour.

"Mr.——, being convinced of the disadvantages to the public, attending the present mode of remunerating medical men, by paying them according to the quantity of drugs they send to their patients, and having adopted the plan of charging FIVE SHILLINGS for ADVICE AND MEDICINE, (when employed for servants, three shillings and sixpence,) which is free from this objection, and which has been much approved of by those who have honoured him with their confidence, takes the liberty of making it known to the families resident in this neighbourhood, that they may avail themselves of it, should they think it desirable."

"—— *street*, —— *place*."

We recommend Mr.——, if he should deem it prudent, for the sake of novelty, to make any change in his address, to employ one of the many placard writers with which the town abounds; for, if the value of his medical attendance be estimated by the elegance of his style, his terms will be thought "all too dear."

---

*Subscription for the Family of the late* Dr. NUTTALL. Dr. Nuttall was a very zealous and liberal member of his profession. He was unfortunately cut off before he could make any provision for his family, and his widow and children are now left nearly destitute. Mr. TUCKER, of Howland street, has very kindly made known the distressing fact to the public, and we doubt not that the hand of charity will be extended towards these deserving objects. Subscriptions are received at all the medical booksellers.

---

Mr. J. GREEN, of Great Marlborough street, requests us to state, that he has not authorised any person to travel about the country, in his name, for the purpose of erecting baths or fumigating apparatus.

### METEOROLOGICAL JOURNAL,

By Messrs. HARRIS and Co. Mathematical Instrument Makers, 50, High Holborn.

| *Month.* | *Rain gauge.* | *Moon.* | *Therm.* | | | *Barom.* | | *De Luc's Hygrom.* | | *Winds.* | | *Atmospheric Variation.* | | |
|---|---|---|---|---|---|---|---|---|---|---|---|---|---|---|
| | | | 9 A.M. | MAX. | MIN. | 9 A.M. | 10 P.M. | 9 A.M. | 10 P.M. | 9 A.M. | 10 P.M. | 9 A.M. | 2 P.M. | 10 P.M. |
| Nov. | | | | | | | | | | | | | | |
| 20 | | | 44 | 46 | 40 | 29.79 | 29.76 | 61 | 63 | S | SSW | Foggy | Rain | Cloudy |
| 21 | | | 45 | 51 | 42 | .50 | .55 | 63 | 64 | SSW | SSW | Cloudy | Fine | Rain |
| 22 | .24 | | 50 | 50 | 42 | .61 | .82 | 64 | 63 | WSW | W | Fine | Fine | Fine |
| 23 | | ☽ | 43 | 46 | 36 | 30.01 | 30.11 | 63 | 62 | W | WNW | — | — | — |
| 24 | | | 39 | 41 | 33 | .25 | .26 | 62 | 63 | NE | ESE | Foggy | Foggy | Fine |
| 25 | | | 37 | 40 | 35 | .23 | .23 | 63 | 62 | ESE | ESE | — | Fine | Cloudy |
| 26 | | | 39 | 40 | 36 | 29.96 | 29.78 | 61 | 60 | SE | SE | — | — | — |
| 27 | .. | | 38 | 38 | 35 | .58 | .50 | 60 | 60 | SE | SE | — | — | — |
| 28 | | | 37 | 44 | 38 | .57 | .66 | 64 | 67 | ESE | SE | — | — | Fine |
| 29 | .15 | | 43 | 44 | 43 | .68 | .75 | 68 | 69 | SSE | E | — | Mist | Sleet |
| 30 | | ⊙ | 44 | 46 | 41 | .78 | .83 | 69 | 68 | E | E | Mist | Foggy | Fine |
| Dec. | | | | | | | | | | | | | | |
| 1 | | | 42 | 42 | 39 | .88 | .86 | 68 | 65 | SE | E | Foggy | — | Cloudy |
| 2 | | | 39 | 42 | 38 | .68 | .56 | 64 | 68 | SSE | SE | — | Mist | Cloudy |
| 3 | | | 38 | 39 | 35 | .43 | .50 | 67 | 66 | SE | NE | — | Foggy | — |
| 4 | | | 37 | 40 | 33 | .61 | .68 | 66 | 66 | NE | NE | — | — | Foggy |
| 5 | | | 36 | 41 | 37 | .63 | .22 | 67 | 67 | NE | ESE | — | — | — |
| 6 | | | 42 | 46 | 45 | .03 | .01 | 68 | 68 | SE | SE | — | — | Cloudy |
| 7 | | ☾ | 47 | 47 | 45 | .00 | .09 | 67 | 67 | ESE | E | — | — | — |
| 8 | | | 45 | 46 | 43 | .06 | .03 | 67 | 67 | ENE | ENE | — | — | — |
| 9 | | | 44 | 45 | 42 | 28.81 | 28.91 | 70 | 71 | ENE | NE | F.& R. | — | — |
| 10 | .52 | | 44 | 44 | 36 | .90 | 29.08 | 72 | 70 | N *va.* | WNW | Foggy | Cloudy | Fine |
| 11 | | | 38 | 41 | 35 | 29.16 | .16 | 69 | 68 | W | WNW | Cloudy | Fine | Cloudy |
| 12 | | | 37 | 40 | 30 | .74 | .96 | 65 | 63 | W | NNW | Fine | — | Fine |
| 13 | | | 32 | 41 | 30 | 30.07 | 30.19 | 62 | 61 | NNW | NW | — | — | — |
| 14 | | | 38 | 39 | 36 | .24 | .26 | 61 | 63 | WSW | WNW | Foggy | — | Clondy |
| 15 | | ● | 39 | 40 | 34 | .31 | .22 | 64 | 65 | WNW | WNW | T. Fog | Mist | Rain |
| 16 | .14 | | 36 | 36 | 30 | .22 | .21 | 67 | 67 | E | SE | Foggy | Cloudy | Fine |
| 17 | | | 33 | 36 | 32 | 29.85 | 29.90 | 66 | 67 | W | NE | — | — | — |
| 18 | .16 | | 34 | 34 | 32 | 30.02 | 30.05 | 67 | 67 | N | N | Fine | Fine | Cloudy |
| 19 | | | 37 | 41 | 35 | .05 | 29.90 | 67 | 66 | WNW | W | — | — | — |

The quantity of Rain fallen in November, was 1 inch and 69-100ths.

